# Supplementary material for: Phase-Selective Synthesis of Mo–Ta–C Ternary Nanosheets by Precisely Tailoring Mo/Ta Atom Ratio on Liquid Copper
Source: Nanomaterials (Basel). 2022 Apr 24;12(9):1446. doi: 10.3390/nano12091446 (PMC9102967; doi:10.3390/nano12091446)
Supplement: Supplementary file 1 [file nanomaterials-12-01446-s001.zip › nanomaterials-1644073-supplementary.pdf]

Phase-Selective Synthesis of Mo-Ta-C Ternary Nanosheets by Precisely Tailoring Mo/Ta Atom Ratio on Liquid Copper

Rong TU [1,2,3], Hang YANG [1], Chitengfei Zhang [1,2,*], Baowen Li [1], Qingfang Xu [1], Qizhong LI [1], Meijun YANG [1], Song ZHANG [1]

[1] State Key Laboratory of Advanced Technology for Materials Synthesis and Processing, Wuhan University of Technology, Wuhan 430070, China

[2] Chaozhou Branch of Chemistry and Chemical Engineering Guangdong Laboratory, Chaozhou 521000, China

[3] Wuhan University of Technology Advanced Engineering Technology Research Institute of Zhongshan City, Zhongshan 528400, China

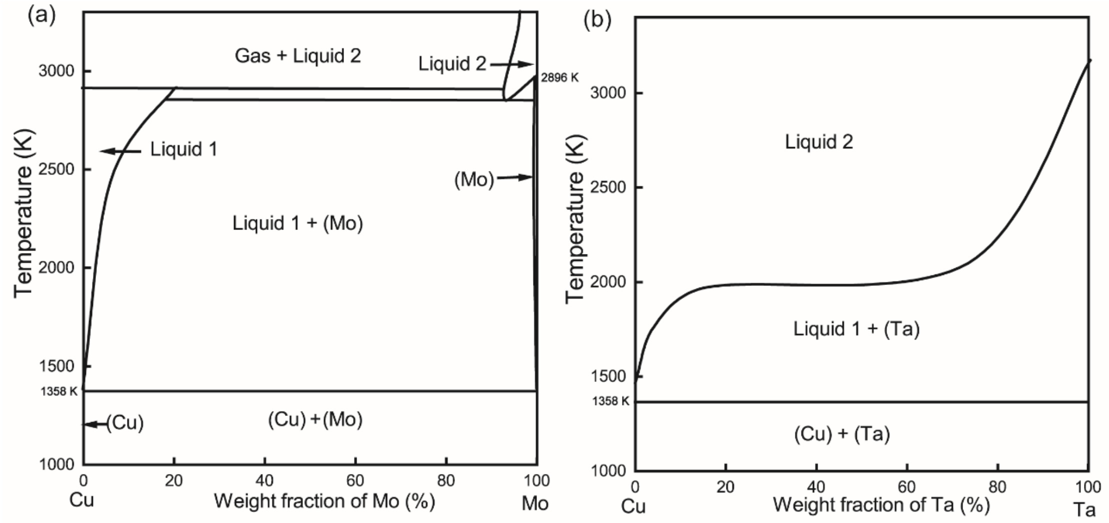

Figure S1. Phase diagrams of Cu-Mo and Cu-Ta.

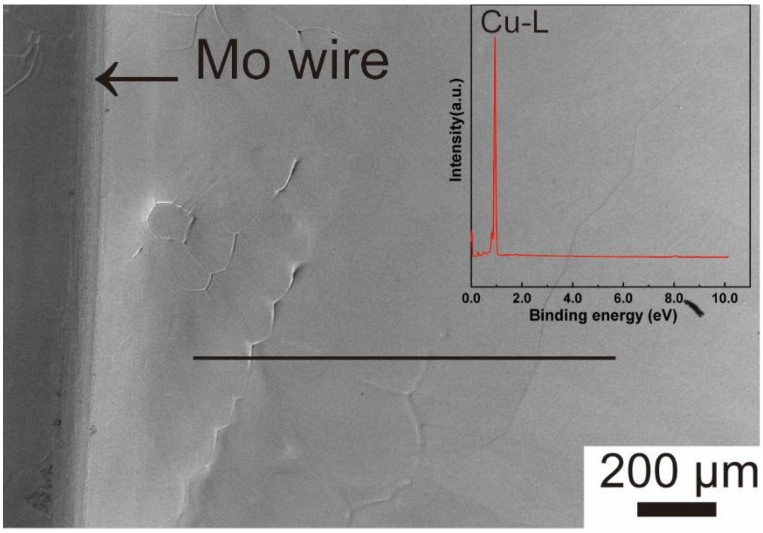

Figure S2. SEM and EDS of Mo wire and Ta foil after diffusion.

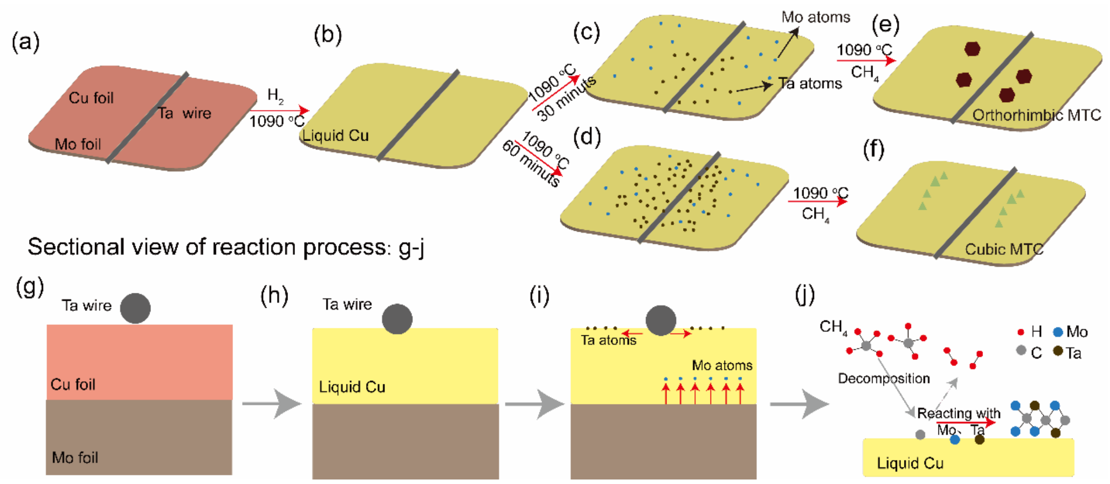

Figure S3. The schematic diagram of orthorhombic and cubic MTC growth processes.

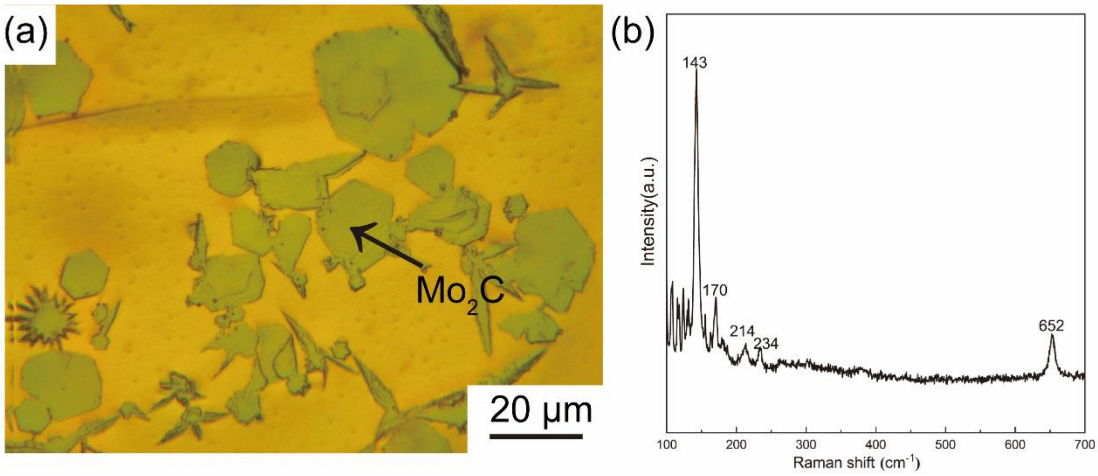

Figure S4. Optical image and Raman of Mo_2C

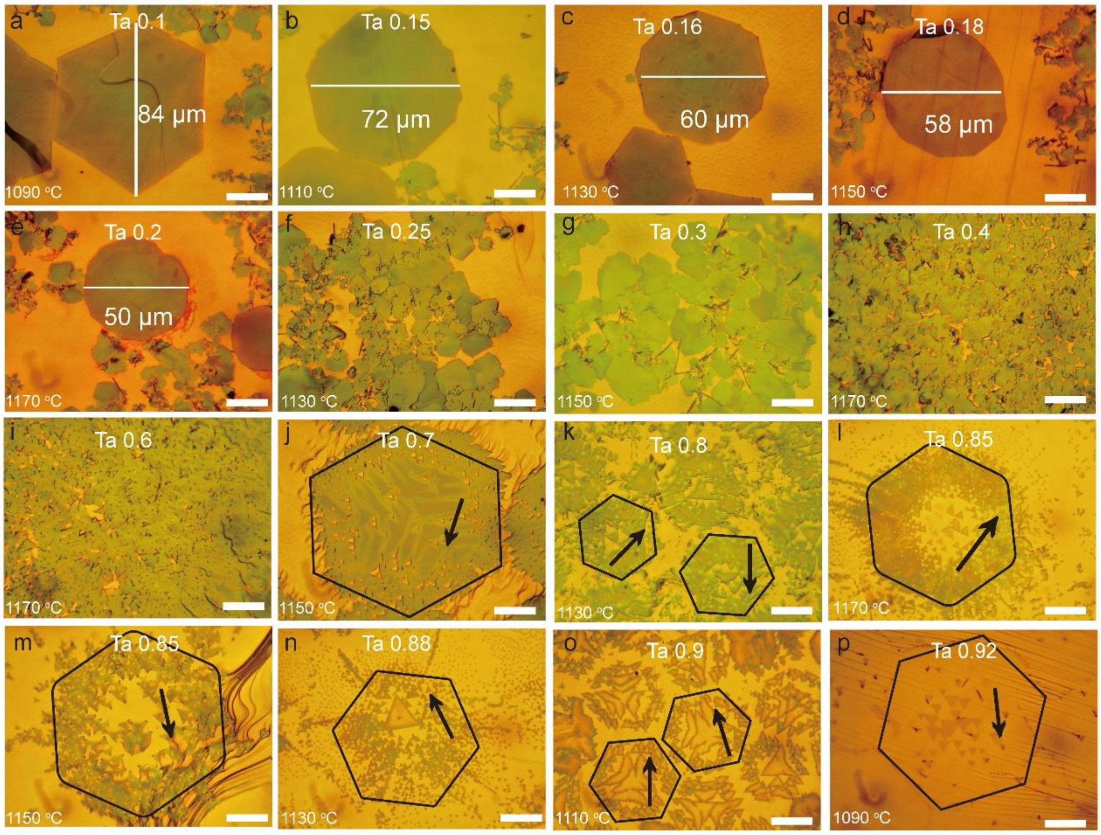

Figure S5. The optical images of orthorhombic and cubic MTC with different Mo/Ta atomic fraction of Ta and temperature. The scale bar is 20 μm.

Table S1. Main characterizations of different synthetic technologies for TMCs

Synthesis method	Precursors and parameters		2D TMC	Size	Thickness	Ref.
	Metal/Carbon sources	Substrate/etching solution				
CVD	Mo foil; CH_4	Cu foil	Mo_2C	~10-100 μm	~3-20 nm	[1]
	Mo foil; CH_4	Cu-Sn foil	Mo_2C	~5 μm	~200 nm	[2]

	Mo foil; CH_4	Au foil	Mo_2C	~10-20 μm	>10 nm	[3]
	V foil; CH_4	Cu foil	VC	~4-77 μm	~10-250 nm	[4]
Chemical Exfoliation	Ta_4AlC_3	50% HF	Ta_4C_3	38 nm	1.325 nm	[5]
	$Hf_3[Al(Si)]_4C_6$	35% HF	Hf_3C_2	200 nm	1.2 nm	[6]
	Ti_3AlC_2	49% HF	Ti_3C_2	2.5 μm	\	[7]
	Ti_3AlC_2	LiF+HCl	Ti_3C_2	5 μm	1.3-400 nm	[8]
	$(Mo_{2/3}Sc_{1/3})_2AlC$	48% HF	$Mo_{1.33}C$	1 μm	\	[9]
	$(V_{0.5}Cr_{0.5})_3AlC$	50% HF	$(V_{0.5}Cr_{0.5})_3C_2$	28 nm	\	[5]
Template	$NH_4)_6Mo_7O_{24}\cdot 4H_2O$; $C_6H_8O_7$	NaCl	Mo_2C	100 nm	20 nm	[10]
	MoO_2 NSs; glucose	\	Mo_2C	~400 nm	~1.0 nm	[11]
Magnetic Sputtering	Mo_2C target	quartz plate substrate	Mo_2C	\	4.3-4.7 nm	[12]
	Nb target; CH_4;	Si (100) substrates	NbC_x	3.9-40.6 nm	2 μm	[13]
CVD	Mo foil; Ta wire; CH_4	Cu foil; anneal 30 min	$(Mo_{2/3}Ta_{1/3})_2C$	84 μm	21 nm	This work
	Mo foil; Ta wire; CH_4	Cu foil; anneal 60 min	$(Mo_{0.13}Ta_{0.87})C$	18 μm	4 nm	This work

References:

1. Xu, C.; Wang, L.B.; Liu, Z.B.; Chen, L.; Guo J.K.; Kang, N.; Ma, X.L.; Cheng,

H.M.; Ren, W.C. Large-area high-quality 2D ultrathin Mo_2C superconducting crystals, *Nat. Mater*. **2015**, *14*, 1135-1141, https://doi.org/10.1038/nmat4374.

2. Chaitoglou, S.; Giannakopoulou, T.; Speliotis, T.; Speliotis, A.; Vavouliotis, A.; Trapalis, C.; Dimoulas, A. Mo_2C/graphene heterostructures: low temperature chemical vapor deposition on liquid bimetallic Sn-Cu and hydrogen evolution reaction electrocatalytic properties, *Nanotechnology*, **2019**, *30*, 125401. https://doi.org/10.1088/1361-6528/aaf9e8.
3. Sun, W.; Wang, X.; Feng, J.; Li, T.; Huan, Y.; Qiao, J.; Ma, D. Controlled synthesis of 2D Mo_2C/graphene heterostructure on liquid Au substrates as enhanced electrocatalytic electrodes, *Nanotechnology*, **2019**, *30*, 385601, https://doi.org/10.1088/1361-6528/ab2c0d.
4. Zhang, C.; Wang, Z.G.; Tu, R.; Dong, M.D.; Li, J.; Yang, M.J.; Li, Q.Z.; Shi, J.; Li, H.W.; Ohmori, H.; et al. Growth of self-aligned single-crystal vanadium carbide nanosheets with a controllable thickness on a unique staked metal substrate. *Appl. Surf. Sci*. **2020**, *499*, 143998, https://doi.org /10.1016/j.apsusc.2019.143998.
5. Naguib, M.; Mashtalir, O.; Carle, J.; Presser, V.; Lu, J.; Hultman, L.; Gogotsi, Y.; Barsoum, M.W. Two-dimensional transition metal carbides, *ACS nano*, **2012**, *6*, 1322-1331, https:// doi.org/ 10.1021/nn204153h.
6. Zhou, J.; Zha, X.; Zhou, X.; Chen, X.; Gao, X.; Wang, S.; Shen, C.; Chen,T.; Zhi, C.; Eklund, P.; et al. Synthesis and electrochemical properties of two-dimensional hafnium carbide, *ACS nano*, **2017**, *11*, 3841-3850. https://doi.org/10.1021 /acsnano.7b00030.

7. Shuck, C.E.; Sarycheva, A.; Anayee, M.; Levitt, A.; Zhu, Y.; Uzun, S.; Gogotsi, Y. Scalable Synthesis of $Ti_3C_2T_x$ MXene. *Adv Eng Mater*, **2020**, *22*, 1901241. https://doi.org/10.1002 /adem.201901241.

8. Maleski, K.; Ren, C.E.; Zhao, M.Q.; Anasori, B.; Gogotsi, Y. Size-dependent physical and electrochemical properties of two-dimensional MXene flakes, *ACS Appl. Mater. Interfaces* **2018**, *10*, 24491-24498, https://doi.org/10.1021/ acsami.8b04662.

9. Tao, Q.; Dahlqvist, M.; Lu, J.; Kota, S.; Meshkian, R.; Halim, J.; Palisaitis, J.; Hultman, L.; Barsoum, M.; Persson, P.; et al. Two-dimensional $Mo_{1.33}C$ MXene with divacancy ordering prepared from parent 3D laminate with in-plane chemical ordering, *Nat. Commun.*, **2017**, *8*, 1-7. https://doi.org/10.1038/ncomms14949.

10. Wu, C.; Li, J. Unique Hierarchical Mo_2C/C Nanosheet Hybrids as Active Electrocatalyst for Hydrogen Evolution Reaction. *ACS Appl. Mater. Interfaces*, **2017**, *9*, 41314–41322, https:// doi.org/10.1021/acsami.7b13822.

11. Jia, J.; Xiong, T.; Zhao, L.; Wang, F.; Liu, H.; Hu, R.; Chen, S. Ultrathin N-Doped Mo_2C Nanosheets with Exposed Active Sites as Efficient Electrocatalyst for Hydrogen Evolution Reactions. *ACS nano*, **2017**, *11*, 12509–12518. https://doi.org/10.1021/acsnano.7b06607.

12. Wang, J.; Liu, S.; Wang, Y.; Wang, T.; Shang, S.; Ren, W. Magnetron-sputtering deposited molybdenum carbide MXene thin films as saturable absorber for passively Q-switched laser. *J. Mater. Chem. C*. **2020**, *8*, 1608-1613. https://doi.org/10.1039/c9tc06117g.

13. Zhang, K.; Wen, M.; Cheng, G.; Li, X.; Meng, Q.N.; Lian, J.S.; Zheng, W.T. Reactive magnetron sputtering deposition and characterization of niobium carbide films. *Vacuum*, **2014**, *99*, 233–241. https://doi.org /10.1016/j.vacuum.2013.06.012.
